# Fasting Ramadan in Chronic Kidney Disease (CKD), Kidney Transplant and Dialysis Patients: Review and Update

**DOI:** 10.7759/cureus.25269

**Published:** 2022-05-24

**Authors:** Elmukhtar Habas, Mehdi Errayes, Eshrak Habas, Khalifa L Farfar, Gamal Alfitori, Ala E Habas, Amnna Rayani, Abdel-Naser Y Elzouki

**Affiliations:** 1 Internal Medicine, Hamad General Hospital, Doha, QAT; 2 Internal Medicine, Tripoli University, Tripoli, LBY; 3 Internal Medicine, Alwakar General Hospital, Doha, QAT; 4 Psychology, Tripoli University, Tripoli, LBY; 5 Hemat-Oncology, Tripoli Pediatric Hospital, Pediatric Tripoli Hospital, Tripoli University, Tripoli, LBY; 6 General Internal Medicine, Hamad General Hospital, Doha, QAT

**Keywords:** kidney transplantation and ramadan, dialysis and fasting, dialysis and fasting ramadan, ckd patients and ramadan, fasting and ckd, ramadan

## Abstract

Chronic kidney disease (CKD) is a common disease in the Islamic regions. Dehydration occurs after prolonged fasting, particularly in hot and humid climates. In the Arabic months’ calendar, Ramadan is a month of maximum given deeds, where Muslims are required to fast from dawn till sunset. Depending on where you live and when the Ramadan month falls, fasting might last anywhere from 10 to 20 hours or more. In certain circumstances, such as poorly controlled diabetes and advanced CKD patients who are allowed to break their fast, the Ramadan fasting amendment is viable. Some Muslims, however, continue fasting despite these circumstances, placing themselves at risk, which is not allowed in the Islamic religion.

There are no medical recommendations that specify who should and should not fast. Nonetheless, the recommendations have been extracted from several published studies. The authors searched EMBASE, PubMed, Google Scholar, and Google for publications, research, and reviews. All authors debate and analyze the related articles. Each author was assigned a part or two of the topics to read, study, and summarize before creating the final draft of their given section. Then this comprehensive review was completed after discussion sessions.

In conclusion, by the Islamic religion view, fasting Ramadan is mandatory for every wise adult person. People who have chronic diseases or that may deteriorate by fasting are exempted from fasting. It seems that fasting and the associated disease hours are determinant factors to fasting or not fasting. Up to our knowledge, there are no established guidelines for CKD patients and physicians to follow; however, the International Diabetes Federation and Diabetes and Ramadan (IDF-DAR) Practical Guidelines 2021 have been issued for CKD diabetic patients and fasting.

## Introduction and background

Chronic kidney disease (CKD) is a prevalent disease globally and increasing every year [1]. CKD is associated mainly with diabetes mellitus (DM) and hypertension (HTN); however, infections such as immune deficiency virus (HIV), malaria, and recurrent urinary tract infection may cause CKD [2]. Comorbid and death rates increase with the progressive reduction of the glomerular filtration rate (GFR) [3]. There was an increase in the mortality rate by about 25% between 2005 and 2015 from renal disease [3]. However, renal disease-induced death may be higher because some countries lack accurate epidemiological data on CKD and inappropriate CKD laboratory-based diagnostic data. A considerable number of CKD patients progress to end-stage renal disease (ESRD), increasing the cost of care by about 1%-3% [4]. It was estimated that more than 5 million patients will require hemodialysis (HD) by 2030 worldwide [5]. Globally, DM is the most common precipitating cause of urinary tract infection, pyelonephritis, and other renal complications such as CKD. Furthermore, DM causes microvascular and cardiovascular complications that may cause CKD. Diabetes is a cause of kidney failure in about half of the patients having renal replacement therapies [6].

Currently, there are more than 2 billion Muslims worldwide. Most of them live in Northern and Central Africa, South coast Asia, and the Middle East. About 1.9 billion adult Muslims worldwide were fasting Ramadan [7], while Puri-Mirza reported that only 1.6 billion fastened 2020 Ramadan [8]. During Ramadan, fasting hours vary between regions, even in the same country, depending on the time of sunrise and sunset. For example, Muslims who are in the habitats of the world’s southernmost countries such as Chile or New Zealand fast for 11 hours on average, while those in northern countries such as Iceland or Norway will be fast for 18 hours or more in some years. Although Muslims who have chronic diseases and use regular medications may harm their health by fasting; hence, these patients can abstain from Ramadan fasting [6,9]. Despite that, some patients still insist on fasting Ramadan, even during the long days of Ramadan. These patients challenge their treating physicians because they are usually multimorbid and need multidisciplinary care. CKD patients require supervision with clear, strict instructions on breaking the fast in case of any deterioration, although some CKD patients insist on continuing to practice fasting [6]. Due to these challenges and problems in CKD patients during fasting, there is no single guideline published for CKD and fasting. However, the International Diabetes Federation and Diabetes and Ramadan (IDF-DAR) Practical Guidelines 2021 is the only recognized guideline on fasting-related DM complication prevention in diabetes and CKD patients [9].

Few large prospective studies investigated the impact of long hours of fasting on CKD. This comprehensive review article will account for the published effects of fasting on CKD, kidney transplant, and dialysis-dependent ESRD patients. To achieve the aim of this review, the authors searched the published articles in EMBASE, Google Scholar, and PubMed using different texts and phrases. Phrases such as “CKD and Ramadan Fasting,” “CKD and longtime absentee of food and drinking fluid,” Ramadan fasting effect on CKD,” dialysis and fasting Ramadan,” “Kidney transplantation and Ramadan fasting,” and “Islamic religion rules in fasting exemptions,” “who should fast and who should not be fast in Islam,” “dehydration effect on CKD,” “impact of water drinking restriction in Ramadan in CKD patients,” and “Ramadan fasting and medications” in CKD were used. There are other keywords and combinations such as “Ramadan,” “fasting,” “kidney,” “dialysis,” “kidney/renal transplantation,” and “chronic kidney disease” in various combinations. All related articles were downloaded and carefully read. Each author has a signed section. Then, after writing a summary of the sections, multiple discussion sessions were done, and then the final manuscript was written.

## Review

Ramadan fasting in Islamic religion

The Islamic calendar is lunar, making the Islamic year has only 354 days instead of 365 days as in the Gregorian solar calendar. This difference between the two calendars might endure Ramadan 29 or 30 days, starting 11 days earlier every year, making Ramadan fall in the cold or hot season. Ramadan fasting is compulsory for each Muslim who is an adult (after puberty), healthy, and able to fast from dawn up to sunset time. Fasting lasts 12-14 hours on average but can last up to 18 hours or even 22 hours at high latitudes [10].

Ramadan fasting is considered an honored act by all Muslims. This honorary act makes even the sick exempted Muslims risk their life for charitable deeds [11]. They start fasting after a pre-dawn meal known as Suhoor, up to the post-sunset time, breaking the fasting at sunset (Iftar). Longtime fasting leads to changes in lifestyle, sleep pattern, and meal intake time, resulting in some endocrine process disturbances and increasing dehydration risk [11]. Besides other exemptions from Ramadan fasting, CKD patients are also exempted, especially when their doctors have concerns about the CKD deterioration due to fasting [9].

Epidemiology of CKD in Muslim Countries

Like other territories, the Arab world has a high prevalence of DM and HTN as the leading underlying cause of CKD. North and Eastern African countries are the second growing prevalent rate area of DM, and it was expected by 2035, DM will increase by 96.2% [10]. In the high-income Middle Eastern countries, the average DM prevalence was 25.4%, whereas, in Gulf Cooperation Council, the prevalence was 12.69% [11]. In other Muslim areas, the estimated prevalence of DM varies. For example, in Pakistan, 17%, Malaysia 20.8%, Indonesia 7%, and Bangladesh 7.4%, forecasted to increase to 13% by 2030 [11-13]. HTN is another causative factor of CKD, increasing the risk of CKD development and progression [14]. The prevalence of HTN in Muslim Middle Eastern countries increased [15]. The increase in HTN and DM prevalence in Muslim countries has led to an increase in CKD prevalence [16]. It was reported that the death rate increased by 179% in 2015 compared to 1990 from diabetes-related CKD [3]. In Saudi Arabia, CKD prevalence was about 5% [17]. Bangladesh has the highest overall prevalence of CKD [18], and renal insufficiency in CKD stages 2-5 patients did not differ significantly between 2006 and 2015 (19% and 17%), respectively [19]. On the other hand, in Iran, CKD prevalence increased by 2.2% from 2011 to 2016 [20]. In Malaysia, CKD prevalence was 9.1% in 2011 [21], increasing expenditure [22], as in most of the other Muslim-majority countries [23].

Guidelines for fasting in CKD patients

DM is a common cause of CKD. Most published guidelines concern DM complications such as hypoglycemia, diabetic ketoacidosis, and hyperglycemia rather than the renal function changes in CKD patients. It is well known that the estimated GFR (EGFR) progressive reduction causes low insulin daily requirement and oral hypoglycemic drug doses [24].

The kidneys degrade about 33% of the exogenous insulin. Insulin filters through the glomerulus and is then reabsorbed by the proximal convoluted tubule. About 60% of insulin renal clearance is due to glomerular filtration, and the other 40% is due to the secretion by uptake from peritubular vessels. Progressive GFR reduction leads to less insulin filtration, which to some extent may compensate partially for the counterbalanced insulin secretion [25], leading to 25% of insulin requirement reduction when the eGFR is between 10-50 mL/min/1.73m^2^ and 50% when eGFR is < 10 mL/min/1.73m^2^ [26]. In ESRD HD-dependent patients with type 2 DM, insulin dose reduction by 26% on the day of the HD session is associated with fewer hypoglycemia symptoms and reasonable glycemic control [27]. Recently, it was reported that advanced CKD and insulin therapy are risk factors in type 2 DM patients, and the risk of severe hypoglycemia is not uncommon [28]. Therefore, it is being advised that CKD patients, especially those with advanced diseases, should check their blood sugar regularly and break their fasting if any harm is expected or observed. They may stop fasting for good after consulting their treating physicians. The guidelines documented that people have DM with advanced CKD are classified as high-risk patients and should not fast [29]. Up to our knowledge, there are no internal or national guidelines for fasting in CKD patients during Ramadan.

Bragazzi published advice for CKD patients who want to fast Ramadan based on a meta-analysis review of the available information in 2014 [30]. In that review, proposed precautions for CKD patients were recommended during Ramadan fasting. The essential precautions are; a) fasting should be broken if serum creatinine increased by ≥ 30% of the baseline, and when hypo/hypernatremia or hyper/hypokalemia symptoms occur. b) Patients must be assessed at least once weekly, and patients must be aware of symptoms of an increase in weight (>2 kg from the baseline), shortness of breath, anorexia, edema, or weakness. c) foods containing high phosphorous and potassium must be avoided, and excessive plain water hydration is essential [30].

CKD and Fasting Ramadan 

previous studies that assessed the effect of fasting on renal function changes during fasting in CKD patients are few, and the studies were either small or uncontrolled. A study assisting Ramadan fasting effect on the renal function parameters in 15 non-dialyzed CKD stage 3b patients with an average GFR of 33 mL/min/1.73 m^2^ was conducted. The study noted that there were no significant differences in the kidney function parameters between the patients and the control group. However, the tubular cell damage marker (N-acetyl-B-D-glucosaminidase) was raised in the urine of the fasted group, indicating the possibility of the Ramadan fasting effect. However, due to the few numbers of fasted patients and the small control group, the investigators had recommended more extensive studies to draw more precise conclusions [31]. Another study was conducted on 60 CKD patients who fasted for the whole Ramadan month. The investigators concluded that 11.7% of the patients had an increase of serum creatinine by 442.1 μmol/L on the initial serum creatinine level, and a 25% reduction of GFR, indicating acute renal failure (ARF) development [32]. However, the authors admitted that the sample size was small, and larger prospective studies are required. Furthermore, Al Muhanna presented data from severe CKD (GFR < 35 mL/min/1.73 m^2^) in 36 patients, which revealed a further significant reduction of GFR [33]. A study of 106 CKD patients with an eGFR of 27.7 mL/min/1.73 m^2^ who fasted Ramadan compared with other severe CKD patients group had an eGFR of 21.5 mL/min/1.73 m^2^ not fast showed significant adverse effects in the fasted CKD patients. In addition to the cardiovascular events and the peripheral vascular disease events, serum creatinine increased by 60.4% after one week of fasting [34]. After three months from the end of Ramadan, the same study reported that plasma creatinine stayed high in 23% of the fasting CKD patients. However, the creatinine rise was not significantly different when compared with the non-fasting CKD patients’ control group, suggesting that the increased creatinine was possibly due to CKD progression rather than fasting [34]. Additionally, a prospective study of 65 CKD (stage 3) patients demonstrated an increase of serum creatinine by ≥ 26.5 μmol/L in 33% of patients [35].

In contrast to the studies mentioned above, other studies reported improvement in renal function during fasting [36-39]. A prospective study observed in 31 CKD patients a significant statistical improvement in the eGFR (from 29.7 mL/min/1.73 m^2^ to 32.7 mL/min/1.73 m^2^ after fasting) in diabetic patients [39]. Hassan et al. documented that despite the insufficient hydration accompanied by reduced serum basal B-type natriuretic peptide, there was no significant eGFR difference between fasting and non-fasting CKD patients with stages 2-4 [38]. Similarly, Turkish patients were studied (45 fastings and 49 non-fastings CKD stages 3-5). The study observed no significant eGFR differences between non-fasting and fasting patients; however, patients aged > 72 years appeared to have a greater risk of renal function deterioration than CKD patients aged < 64 years [37]. A Saudi Arabia-based clinical study of 39 patients with CKD stages 3 and 4 revealed no significant differences in clinical and laboratory parameters during fasting [36]. A study of diabetic kidney disease patients who fasted for about 19 hours during the summer of 2018 reported a significant difference in proteinuria and ARF risk in 68 CKD stage 3 diabetic patients compared with 61 same category patients who did not fast [40]. A retrospective study found that in 1199 patients who had not been exempted from Ramadan fasting for two years (2016 and 2017). The study found that fasting significantly reduced the risk of developing ARF, especially in patients with comorbid conditions, indicating Ramadan fasting has no negative effects on most patients with comorbid conditions. However, they recommended other prospective bigger studies to confirm their conclusion [41]. In studied CKD patients, the eGFR was not changed significantly during Ramadan in cold seasons, and patients can fast with no complications as long as the follow-up is regular. However, the author of this review recommended further extensive larger studies should be conducted [30]. Ekinci et al. reported no significant deterioration of renal function in autosomal dominant polycystic disease patients with early CKD stage following Ramadan fasting [42].

Bernieh et al. showed a reduction in urinary protein and sodium excretion and concluded that the studied CKD patients had good tolerance and safety of fasting Ramadan [39]. Another prospective cohort study of CKD grades 2-4 revealed a significant increase in serum urea levels (p = 0.024) during the last seven days of Ramadan, which returned to basal levels after the following month. On the other hand, the eGFR did not significantly alter at the end of Ramadan, and the plasma B-type natriuretic peptide levels reduced significantly after fasting (p ≤ 0.021), which returned to basal values four weeks later [38]. Baloglu et al.'s study revealed that HTN and fasting days are significant predictive risks for ARF. Out of 117 with an average of 60 years with stages 2-3 CKD patients, 27 patients developed ARF. They concluded that good hydration and regular check-up could reduce ARF risk [43]. A recent Egyptian study reported that serum creatinine raised significantly after fasting Ramadan, and the eGFR decreased insignificantly in patients without CKD. On the contrary, serum creatinine in CKD patients was reduced, and eGFR improved significantly, most probably due to improved blood pressure control in hypertensive patients with CKD [44].

It is difficult to speculate the explanations for the disparities observed between the different research discussed previously. The study populations are highly heterogeneous in CKD severity and the fasted days, fasting duration, and observation period. However, in research involving individuals with greater degrees of renal impairment, it appears as though fasting is more detrimental, but this is not always true in the aforementioned articles. Table 1 provides a summary of the above cited literature for fasting and CKD.

**Table 1:** Published articles summary of CKD and Ramadan fasting Chronic Kidney Disease (CKD), Estimated Glomerular Filtration Rate (eGFR), Hypertension (HTN), Acute Renal Failure (ARF)

Authors/Year	Kidney serum parameters	Remarks	Conclusion
Bragazzi [29]/ (2015)	No significant reduction in eGFR	Cold weather does not improve eGFR	More studies required
El-Wakil et al., [31]/ (2007)	No significant difference	Increased tubule markers in urine of fasting group	Larger prospective studies recommended
Al Muhanna [33]/ (1998)	eGFR reduction	Possibility of renal deterioration	Larger studies were recommended
NasrAllah & Osman [34]/(2014)	eGFR reduction	Increased risk of CKD deterioration with significant increase of serum creatinine in the fasting group	Deterioration was possibly due to CKD progression, no fasting
Bakhit et al. [35]/ (2017)	Increase in creatinine by ≥ 26.5 μmol/L	Increase of serum creatine in 33% of patients (prospective)	Fasting affects CKD patients and further studies are required
Al Wakeel [36]/(2014)	No significant differences in clinical and laboratory parameters	CKD stages 3 and 4 patients	Fasting has no significant effect
Kara et al. [37]/(2017)	No significant eGFR difference	Increased risk of eGFR reduction in patients aged > 72 years	Age related CKD deterioration or dehydration risk
Hassan et al. [38]/(2018)	Increased urea levels, improved after Ramadan. The eGFR rate was not altered, BNP levels significantly reduced	CKD grades 2-4 patient, eGFR not effectively changed (prospective)	CKD grades 2-4 can fast with a reasonable degree of safety.
Bernieh et al. [39]/(2010)	eGFR significantly improvement during Ramadan & the month after	No significant change of CKD progression, reduced proteinuria & sodium urine excretion (prospective)	Good tolerance and safety of fasting, good diet control, regular follow up and encourage water drinking
Chowdhury et al. [40]/ (2019)	Significant increase of proteinuria and the risk of acute renal failure	CKD stage 3 diabetic patients	Fasting has a risk of ARF and CKD progression, regular close monitoring
AlAbdan et al. [41]/ (2022)	No change in renal function parameters during fasting, even in comorbid patients	Significant reduction of ARF risk in patients with comorbid diseases	Larger prospective studies were advised to investigate the beneficial effect of fasting in ARF reduction
Ekinci et al. [42]/ (2018)	No significant deterioration of renal function	Proteinuria is significantly improved	In adult autosomal dominant polycystic disease patients with early CKD stage following Ramadan fasting
Baloglu et al. [43]/(2020)	Significant increase of serum urea	About 23% of the CKD stage 2-3 patients had ARF. A significant link between HTN, the number of fasting days, ARF. (prospective)	Patients CKD stage 2-3 and HTN must be evaluated more carefully, encouraging well hydration, and strictly followed for ARF
Eldeeb et al. [44]/(2020)	Improved eGFR and serum creatinine	In stage 3-4 CKD patients with HTN, central and blood pressure improved (prospective)	Fasting improves blood pressure control and renal function

Fasting in people with renal transplant 

Multiple studies were conducted to investigate the impact of fasting on kidney function in renal transplanted patients. A study conducted in Saudi Arabia examined thirty-five renal transplant recipients who fasted for three months (a month before Ramadan, Ramadan, and one month after Ramadan). There were no significant differences in eGFR (56.4 mL/min/1.73 m^2^ versus 55.4 mL/min/1.73 m^2^ and proteinuria, respectively, between these patients and the control group who did not fast for the same period [45]. Another study of 23 kidney transplanted patients reported no significant difference in blood and urine renal function parameters after fasting in the transplanted kidney patients with normal parameters and stable higher renal parameters [46]. A study that matched 19 fasting patients with 20 control kidney transplanted patients revealed that the serum creatinine had no significant difference before and after fasting Ramadan [47]. Furthermore, when 41 fasting renal transplant patients were compared with 41 non-fasting kidney transplant patients, no significant change in serum creatinine levels was observed, even in patients with moderate CKD post-transplant [48]. An Iranian study of 30 patients with kidney transplantation found that their creatinine and electrolyte levels stayed steady during and after Ramadan [49].

Said et al. investigated 145 kidney transplanted patients with serum creatinine < 200 µmol/L and showed no significant difference between non-fasting and fasting groups in serum creatinine concentration changes [50]. A prospective matched case-control observational study reported an identical conclusion. The study compared 43 fasting with 37 non-fasting renal transplant patients during hot weather in Riyadh. The eGFR and plasma creatinine levels showed no difference before and after Ramadan fasting. On subgroup analysis, eGFR changes were not significantly different in patients with eGFR < 45 mL/min/1.73 m^2^ and those who had eGFR 45-75 mL/min/1.73 m^2^ before and after 19.6 ± 1.3 months of Ramadan fasting [51]. Two years of follow-up of kidney transplanted patients gave almost a similar conclusion [51]. A study of 43 kidney transplant patients with an eGFR of > 75 mL/min/1.73 m^2^ reported no significant difference between the fasted and the non-fasted participants, and fasting in the month of Ramadan during the hottest months in two consecutive years [52]. In a study of 14 kidney transplanted patients who had the transplantation <12 months before Ramadan and had a mean serum creatinine of 115 μmol/L, their serum creatinine remained stable before and after Ramadan fasting, but serum urea increased significantly [53]. Another study was carried out also in Riyadh, Saudi Arabia, for fasted 280 kidney transplant patients compared with 285 non-fasting renal transplant patients during the Ramadan period. The comparison had not shown a significant difference in eGFR between the two groups, and the eGFR stayed stable with an average of 72 mL/min/1.73 m^2^ [54]. A meta-analysis review about fasting in CKD kidney transplanted patients concluded that fasting did not affect kidney function parameters [30]. Another systemic review noted that the incidence of renal colic increased following fasting in CKD and kidney transplanted patients, and documented fasting was well tolerated [55]. Table 2 summarizes the above-mentioned studies on fasting and kidney transplantation.

**Table 2:** Published articles summary of kidney transplantation and Ramadan fasting Estimated Glomerular Filtration Rate (eGFR)

Authors/Year	Remarks	Conclusion
Bragazzi [30]/2014	Me-tanalysis review, no effect of fasting on eGFR	Able to fast
Ghalib et al. [45]/(2008)	35 patients, eGFR and proteinuria were not different significantly	Able to fast
Abdalla et al. [46]/(1998)	23 patients, stable renal function parameters	Able to fast Ramadan
Einollahi et al. [47]/(2005)	19 patients, serum creatinine did not change significantly	Able to fast
Einollahi [48]/(2009)	41 patients, no significant change of serum creatinine even in high creatinine baseline value patients	Able to fast
Argani et al. [49]/(2003)	30 patients, serum electrolyte and creatinine levels remained stable	No significant effect of fasting
Said T et al. [50]/(2003)	145 patients, no significant changes in serum creatinine	No effect of fasting, able to fast
Hejaili et al. [51]/(2014)	43 patients, no significant change in eGFR and serum creatinine. No difference even in 2 years follow up	Able to fast
Qurashi et al. [52]/(2012)	43 Patients, no significant difference between the fasted and the non-fasted, and the month of Ramadan fasting in two consecutive years during the hottest months	Able to fast
Ouziala [53]/1998	14 patients, serum creatinine stable, but urea increased.	Able to fast, good hydration and regular follow up
Ibrahim et al. [54]/2018	280 patients, no difference in eGFR	Able to fast
Bragazzi [55]/2015	Systematic review, Increase incidence of renal colic	Fasting was well tolerated

Fasting in dialysis-dependent ESRD patients 

Al Wakeel et al. studied patients on peritoneal dialysis (PD) who fasted during Ramadan for almost 14 hours daily. He noticed no adverse effects of fasting on these patients [56]. A study of 41 HD-dependent ESRD patients who started HD at a minimum for six months revealed no significant change in weight, blood pressure, and serum potassium [57]. A comparative study of 34 fasting HD patients and 252 non-fasting HD patients reported no difference in the harm risk of fasting and no increase in the death or morbidity rate between the fasting group. However, serum albumin and phosphorus increased in the fasting group compared to the non-fasting group [58]. Wan et al. had also studied HD-dependent 35 patients who were 50% of them were diabetic and had fasted Ramadan. They concluded no weight changes, and the serum phosphorous and albumin levels were improved [59]. A multicentric observational prospective study in Saudi was 635 HD-dependent patients involved. The fasting group that represented the two-third of the patients had only a mild increase in phosphorus serum level, though it was statistically significant (2.78 ± 1.8 versus 2.45 ± 1.6 μmol/L; p = 0.045) [60].

In contrast to the studies cited earlier, Alshamsi et al. study results had not shown any significant differences before or after HD in blood pressure, weight gain, death or morbidity, serum albumin, phosphorus, or other elements [60]. Another multicenter study that looked at 68 HD-dependent ESRD patients in Malaysia after 20 days of fasting found that body mass index, intradialytic weight, and serum creatinine, urea, and phosphate levels were all improved whereas serum albumin levels dropped. Additionally, they concluded that intermittent Ramadan fasting changes nutritional status parameters for a short time; however, the changes had no long-term adverse effects on patients who are on long-term HD treatment [61].

A more extended period study over 24 years of 1,841 HD patients in Pakistan reported an increase in the death rate among 897 registered patients (48.7%) during Ramadan. The results of this study reported a higher rate of death during Ramadan than in other months (11%). However, there was a lack of fasting evidence that had not permitted firm conclusions by the authors on whether the increase is related to fasting or the other comorbidities [62]. In another study involving 32 HD-dependent patients who fasted during Ramadan, the serum levels of creatinine, urea, phosphorus, uric acid, and red blood cell count increased significantly. Hyperkalemia and hypernatremia occurred in 15.6% and 25% of patients, but none of these complications necessitated hospitalization [36]. Although there are some reports that fasting might be harmful to some HD-dependent patients, the overall conclusions suggest that fasting is relatively well-tolerated and does not affect the morbidity and mortality rates. However, careful monitoring of serum electrolytes is advisable, especially for potassium and sodium. The cited aforementioned articles are summarized in Table 3.

**Table 3:** Published articles summary of dialysis and Ramadan fasting Hemodialysis (HD), Peritoneal Dialysis (PD)

Authors/Year	Remarks	Conclusion
Al Wakeel [36]/(2014)	HD, Hypernatremia and hyperkalemia	None required hospitalization
Al Wakeel et al. [56]/(2013)	PD, no adverse effect from fasting	Able to fast Ramadan
Al-Khader et al. [57]/(1991)	HD, no significant effect on weight, BP or serum potassium	Able to fast
Imtiaz et al. [58]/(2016)	HD, no harms effect, death rate does not increase, increased Phosphorus and potassium	Close observation
Wan et al. [59]/(2014)	HD, No weight increase, serum albumin improved	Able to fast, close attention
Alshamsi et al. [60]/(2016)	HD, Significant increase in phosphorus, BP, albumin and weight no change	Able to fast, close follow-up
Adanan et al. [61]/(2020)	HD, improved intradialytic serum phosphorus, creatinine, weight, but albumin decreased	Participants young, need close monitoring
Imtiaz et al. [62]/(2015)	HD, Higher mortality	Possibly death due to comorbid, do not urge patient to fast. Further studies are needed

Recommendations for Ramadan fasting in kidney diseased patients

Ramadan fasting puts CKD patients at a higher risk of complications, especially those with CKD stages 4-5, and they may need to break the fasting for medical or religious exemptions. Furthermore, CKD in patients with cardiovascular disease should not be fast because they may progress quickly. According to research, abrupt loss of renal function in CKD stage 3 patients is possible, and some stable CKD stage 3 patients can fast. They should, however, be cautious and vigilant, and they should be aware that if their health deteriorates, they may need to discontinue fasting.

Fluid drinking at night is advisable but should not be excessive to the extent that it may lead to overload, which is possible to assess by self-daily weight and examination for edema. In cases who have an increase of serum creatinine by 30% from the baseline or a significant change in serum electrolytes levels must be advised to break their fasting. The frequency of creatinine, urea, and electrolytes assessment depends on the local recommendation; however, ten days is a reasonable period in stable CKD patients. Moreover, once or twice weekly monitoring the CKD patient for change in body weight (>2 kg is significant), breathlessness, anorexia, body instability, weakness, or edema. Furthermore, these patients should avoid high contained potassium and phosphorus food.

In kidney transplant patients who have a stable renal function and taking their immunosuppressive can fast Ramadan safely if their physicians regularly follow them before and after Ramadan. However, these patients need to drink enough fluids and take immunosuppressive therapies during the non-fasting period. Some dialysis ESRD-dependent patients can do fasting; however, it is highly recommended for those patients to discourage fasting. If they insist on fasting, they require regular monitoring and should be reviewed carefully on dialysis days.

The IDF-DAR guideline recommendations for patients with CKD, ESRD, and renal transplant are as follows: a) Ramadan fasting is permissible in persons with stable chronic kidney disease or who have undergone a kidney transplant and have temporary biochemical abnormalities. However, this may be true for diabetics, but additional research into pre-existing DM and chronic kidney disease is necessary. b) During Ramadan, patients who have received a kidney transplant or have stages 3-5 chronic renal disease are at a higher risk of worsening. Before Ramadan, these patients require close monitoring and individualized advice. Additionally, the guideline recommends that more considerable prospective studies, including randomized trials and studies examining the effect of fasting on vascular tissues in patients with DM and associated consequences, are necessary. Malik et al. suggested that well-designed observational studies with large sample sizes, or Randomized control trials, be conducted to fill the knowledge gap. They suggested a risk stratification and patient management method, enabling patient-centric conversations, assisting decision-making, improving patient and clinician satisfaction, and providing a safe and secure Ramadan experience [63].

## Conclusions

Fasting overextended longer hours (particularly in a hot climate during summer) increases the risk of ARF and CKD worsening, primarily due to induced dehydration caused by increased perspiration and decreased fluid consumption during Ramadan’s eating time. Although individuals may be exempt from fasting for religious or medical reasons, some persist in fasting, seeking honorable deeds, and placing themselves in danger.

All health practitioners who care for CKD patients should be aware that there are no well-established, documented facts regarding whether to advise CKD patients to conduct fasting or not. However, many observational studies have demonstrated that fasting has no meaningful influence on the deterioration of renal parameters in patients with chronic kidney disease.

One of the factors that help to guide whether to fast or not in CKD patients is the number of fasting hours. Fasting for 12-14 hours or even 20 hours may be possible during the winter, but Ramadan fasting should be carefully considered during the hot months, especially if the fasting exceeds 12 hours. However, in both cases, individualized advice, close monitoring, and follow-up are required.

According to the IDF-DAR guideline, high or extremely high-risk patients are more likely to develop uncontrolled DM and CKD deterioration than one or low-risk groups. However, those patients need close monitoring and will have to break their fasting and are encouraged to consider alternatives to fasting. All patients must receive current education on sick-day policies and when to break their fast or abstain from fasting before Ramadan.

The previous analysis indicates the lack of data available to clinicians to aid in fasting decisions in CKD patients. Consequently, controlled studies comparing CKD patients who are fasting to those who are not fasting are essential for assisting clinicians and patients. Appreciation and formulation of clear guidelines for CKD patients require coordinated multicenter efforts analogous to the DAR group approach to DM treatment during Ramadan, which has established a body of information to aid clinicians in counseling diabetic patients who are fasting.
